# Protein expression based multimarker analysis of breast cancer samples

**DOI:** 10.1186/1471-2407-11-230

**Published:** 2011-06-08

**Authors:** Angela P Presson, Nam K Yoon, Lora Bagryanova, Vei Mah, Mohammad Alavi, Erin L Maresh, Ayyappan K Rajasekaran, Lee Goodglick, David Chia, Steve Horvath

**Affiliations:** 1 Department of Biostatistics, UCLA, Los Angeles, CA, 90095, USA; 2 Department of Pediatrics, David Geffen School of Medicine, UCLA, Los Angeles, CA, 90095, USA; 3 Department of Pathology and Laboratory Medicine, David Geffen School of Medicine, UCLA, Los Angeles, CA, 90095, USA; 4 Department of Human Genetics, David Geffen School of Medicine, UCLA, Los Angeles, CA, 90095, USA; 5 Jonsson Comprehensive Cancer Center, David Geffen School of Medicine, UCLA, Los Angeles, CA 90095, USA; 6 Nemours Center for Childhood Cancer Research, Wilmington, DE, USA

**Keywords:** Tissue microarray, breast cancer, tumor marker, prognostic marker, WGCNA

## Abstract

**Background:**

Tissue microarray (TMA) data are commonly used to validate the prognostic accuracy of tumor markers. For example, breast cancer TMA data have led to the identification of several promising prognostic markers of survival time. Several studies have shown that TMA data can also be used to cluster patients into clinically distinct groups. Here we use breast cancer TMA data to cluster patients into distinct prognostic groups.

**Methods:**

We apply weighted correlation network analysis (WGCNA) to TMA data consisting of 26 putative tumor biomarkers measured on 82 breast cancer patients. Based on this analysis we identify three groups of patients with low (5.4%), moderate (22%) and high (50%) mortality rates, respectively. We then develop a simple threshold rule using a subset of three markers (p53, Na-KATPase-β1, and TGF β receptor II) that can approximately define these mortality groups. We compare the results of this correlation network analysis with results from a standard Cox regression analysis.

**Results:**

We find that the rule-based grouping variable (referred to as WGCNA*) is an independent predictor of survival time. While WGCNA* is based on protein measurements (TMA data), it validated in two independent Affymetrix microarray gene expression data (which measure mRNA abundance). We find that the WGCNA patient groups differed by 35% from mortality groups defined by a more conventional stepwise Cox regression analysis approach.

**Conclusions:**

We show that correlation network methods, which are primarily used to analyze the relationships between gene products, are also useful for analyzing the relationships between patients and for defining distinct patient groups based on TMA data. We identify a rule based on three tumor markers for predicting breast cancer survival outcomes.

## Background

Breast cancer is the most common type of cancer in women. While survival rates are improving, nearly one in eight women is expected to acquire breast cancer. Current knowledge of breast cancer etiology and treatment protocols has benefited from the simultaneous analysis of multiple biomarkers. At the turn of the century, the combination of low estrogen receptor (ER), progesterone receptor (PR) and human epidermal growth factor receptor-2 (HER2) expression levels was shown to identify a high risk "triple-negative" breast cancer phenotype [1,2] that occurs in 10-20% of breast cancers and indicates that the cancer cannot be effectively treated by conventional therapies [3,4]. More recently, a BCL2/FOS gene expression signature was discovered that can delineate breast cancer patients that have poor tamoxifen response [5]; and a Ki67, P53 and GATA3 combination was shown to predict success of hormonal therapy in ER positive patients [6].

High-density breast tissue microarrays (TMA) and proteomics data have been useful for prognosticating cancer outcomes [5,7]. The immunohistochemical staining patterns measured by TMAs allow one to determine the cellular location and intensity of protein expression levels. TMAs facilitate an accurate and high-throughput analysis of archived tumor specimens [8,9] which allows one to analyze hundreds of patients but typically relatively few markers. In comparison, gene expression microarrays require fresh or frozen tissue, but they can assay expression levels (messenger RNA abundances) of thousands of genes simultaneously. Thus, a common workflow is to identify candidate markers using gene expression arrays and then to validate the prognostic accuracy of corresponding protein measures using a TMA platform. Here we reverse the direction of this common workflow. We start with breast cancer TMA markers that evaluate the staining patterns of 26 genes. Next, we use correlation networks to classify patients into distinct survival groups. We develop a prognostic rule (referred to as WGCNA*) based on a subset of markers that can be used to classify patients into distinct survival groups. Finally we validate the prognostic accuracy of this rule in two independent Affymetrix HG-U133A gene expression data sets.

While the methods for identifying a single candidate biomarker for breast cancer prognosis are relatively straightforward, there is a need for simple and effective methods that can jointly analyze multiple biomarkers. Here we propose methodology based on weighted correlation network analysis for the simultaneous analysis of multiple tumor expression array (TMA) markers [10,11]. WGCNA has been used in cancer and mouse genetic studies for analyzing the pairwise relationships between gene expression levels [12-16]. WGCNA has primarily been used to identify genes with similar RNA expression profiles across patients, but here we use WGCNA to define groups of patients that have similar tumor expression profiles across multiple TMA markers. While the nodes of a gene co-expression networks are genes, the nodes of our patient sample networks are breast cancer patients. We use the breast cancer sample network to identify groups of patients that have similar expression profiles, resulting in a "patient network". Second, the patient network is related to survival information to identify cancer subtypes, or WGCNA mortality groups. We then apply classification and regression trees to identify representative TMA markers, p53, Na-KATPase-β1, and TGF β receptor II that best predict these patient subtypes. Finally, we compare our WGCNA method to a traditional step-wise multimarker Cox regression analysis approach (referred to as the "COX" approach).

We show that both WGCNA and the COX approach identify candidate biomarkers that have a significant association with cancer survival time. However, step-wise methods are notorious for over-fitting the data, yielding results that are not reproducible in other data sets. To compare the validation success of our WGCNA markers (p53, Na-KATPase-β1, and TGF β receptor II) with those of the COX approach, we use three independent gene expression data sets. We find that the WGCNA groups and markers have superior validation success.

## Methods

### Breast Tissue Microarray

A high-density breast TMA was constructed using cores from formalin-fixed, paraffin embedded breast tissue donor blocks, consisting of 242 breast surgical cases of 210 patients who underwent surgery at the UCLA Medical Center between 1995 and 2000, as previously described [17,18]. Archival samples were obtained from the UCLA Department of Pathology and Laboratory Medicine with oversight and approval from the UCLA institutional review board. Such samples were consented for use in biomedical research projects at the time of surgery. At least three cores of each available histologic type were arrayed from the donor blocks. Of the 242 surgical cases, 179 cases (from 157 patients) were of invasive breast cancers of various histologic types. For our multimarker analysis, we selected 82 primary surgical cases, each belonging to a unique patient with invasive cancer who did not receive neoadjuvant therapy, had disease-specific survival outcome, and were informative for expression in most of our protein markers.

### Immunohistochemistry

Immunohistochemical staining of the breast TMA was performed using a standard two-step indirect avidin-biotin complex method (Vector Laboratories, Burlingame, CA) or a two-step polymer detection method (DakoCytomation, Inc., Carpinteria, CA) as previously described [19-22]. The following primary antibodies were used: BS106, BU101, Mammaglobin (Abbot Laboratories, Abbott Park, IL), prolactin-inducible protein (Signet Laboratories, Inc., Dedham, MA), S100A7 (Imgenex Corp., San Diego, CA), 14-3-3 σ (Research Diagnostics, Inc., Concord, MA), Her-2/neu (Zymed Laboratories, Inc., South San Francisco, CA), progesterone receptor, estrogen receptor alpha, p53 (DakoCytomation, Inc., Carpinteria, CA), RIN1, annexin A1, beta-catenin (BD Biosciences Transduction Laboratories, Lexington, KY), Na-KATPase-β1, Na-KATPase-α, GATA3, Smad2 (Santa Cruz Biotechnology, Inc., Santa Cruz, CA), Smad4 (Millipore, Billerica, MA), YY1 (Geneka Biotechnology, Inc., Montreal, Quebec, Canada), TGF β receptor II (Abcam, Inc., Cambridge, MA), H3K4 and H3K18 (Upstate, Lake Placid, NY), and MED28 (gift from Dr. Mai Brooks). Briefly, 4 μm sections were deparaffinized, treated with 0.3% hydrogen peroxide in methanol, blocked with 5% serum, and incubated with primary and secondary antibodies. Diaminobenzidine was used for color detection. A concentration-matched isotype control IgG was used for negative controls. Note that the 26 markers analyzed here were originally chosen for other oncogenic studies conducted in our laboratory.

The level of protein expression in glandular epithelial cells was quantitatively assessed by a pathologist blinded to all clinico-pathological variables. We used the percentage of cells staining, referred to as "pos", as the quantitative measure of protein expression. To arrive at a single staining measure per patient (referred to as "pos.mean"), we averaged the pos measures of multiple cancer spots per patient as described in [23].

### Validation data analysis

To validate our WGCNA* and COX mortality group definitions, we selected all Affymetrix HG-U133A gene expression data sets from the Gene Expression Omnibus (GEO) that were published in 2005 or later. This resulted in three independent data sets published in 2005-2006, that had the following GEO identifiers: Miller 2005 - GSE3494 (251 arrays), Pawitan 2005 -GSE1456 (159 arrays), Sotiriou 2006 - GSE2990 (189 arrays) [24-26]. Data sets were pre-processed as described in [27]. Briefly, within each data set we evaluated array quality by comparing inter-array correlations. Arrays with low inter-array correlation were removed according to default recommendations [27]. When expression analysis was distributed across multiple centers, we checked for center-related batch effects. If batch effects were present we removed them using the combat function [28]. The pre-processing steps removed 3-12% of arrays in an unbiased fashion resulting in 222, 146 and 183 arrays for the Miller 2005, Pawitan 2005 and Sotiriou 2006 data sets, respectively. Finally, we removed all samples with missing survival data, resulting in a total of 207, 146 and 173 patients for the Miller, Pawitan and Sotiriou data sets, respectively.

Univariate Cox proportional-hazards models were constructed for the WGCNA* patient groups and COX rule patient groups for each of the three data sets. We used a moderate significance level of 0.1 to allow for expected expression differences between genes and proteins.

## Results

In this section we present steps for conducting a Weighted Correlation Network Analysis (WGCNA) of tumor expression data to identify patient groups that have high, moderate, and low survival. We then present results from applying WGCNA to a breast cancer data set consisting of 26 markers measured on 82 patients. We compare the WGCNA results to a more conventional multimarker analysis approach and then show that the WGCNA results validated in two of three Affymetrix gene expression HG133A array data sets.

### Steps for conducting a Weighted Correlation Network Analysis (WGCNA) of patients

**Figure 1:**
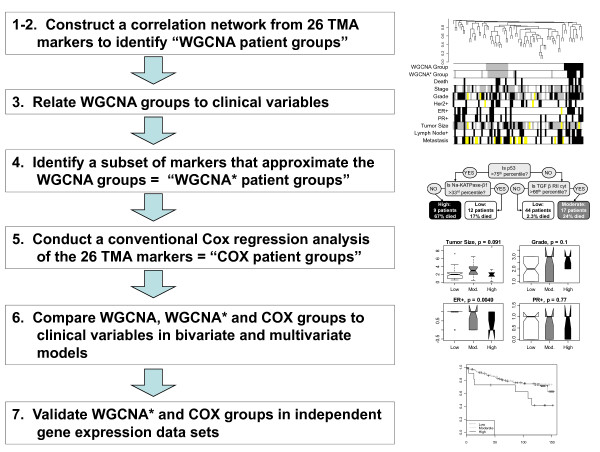
**Overview for conducting a Weighted Correlation Network Analyses (WGCNA) of patient TMA data (Steps 1-4) and follow up analyses (Steps 5-7)**. Steps 1-4 are numbered to correspond with the WGCNA methods section in the text. After defining WGCNA and WGCNA* patient groups, we compare these results to a more conventional variable selection approach (Steps 5-6). Finally, we validate the WGCNA* and conventional results in independent Affymetrix gene expression data sets (Step 7).

In the following, we outline the analysis steps for conducting a WGCNA of the TMA patient data. An overview diagram is provided in Figure 1. R software for WGCNA and accompanying software tutorials are freely available at: http://www.genetics.ucla.edu/labs/horvath/CoexpressionNetwork/.

#### 1. Create a patient correlation network from tumor marker expression data

We used WGCNA to identify clusters of patients whose tumor marker profiles were positively correlated. In this analysis, patients are considered "nodes" of the network, and edges between them are determined by correlations across the set of tumor markers. WGCNA was performed using R software functions (indicated in courier font) provided in the WGCNA R package [10,11,29-31].

There are two types of weighted correlation networks, "unsigned" and "signed". An unsigned network is based on the absolute value of the Pearson correlation coefficient, while the signed network is based on the conventional Pearson correlation coefficient ("cor"). Specifically, the network adjacency (connection) between a pair of samples *x*_*i *_and *x*_*j *_is defined as *a(i,j) *= (0.5 *+ *0.5cor(*x*_*i*_*, x*_*j*_))^ *β *where the power *β *facilitates a soft thresholding approach that emphasizes high positive correlations at the expense of low or negative correlations. We recommend a signed correlation network approach for comparing patient expression profiles since it is unlikely that negatively correlated samples are molecularly similar. In practice, we find that patients have moderate to high positive expression correlations across protein markers. Based on the network adjacency matrix, we define the following dissimilarity measure between the samples: *dissA*= *1-adjacency*. While other network dissimilarities have been used in correlation network analysis (e.g. the topological overlap based measure [32,33]) we recommend *dissA *since it leads to clusters of positively correlated samples.

#### 2. Define patient groups (modules) from the patient network

The sample network dissimilarity *dissA *can be used as input of a clustering procedure. Here we used average linkage hierarchical clustering using the flashClust WGCNA function. Clusters of patients were defined as branches of the resulting cluster tree. To "cut" the branches of the tree, we used the cutreeDynamic R function since it affords more flexibility than traditional approaches and has been carefully evaluated in several simulation studies where it was shown to retrieve the true simulated module structure [34-36]. By construction, the resulting clusters of patients (also referred to as groups or "modules" of patients) have positively correlated expression profiles across the tumor marker set. The molecular profiles of each module can be represented using the first principal component (referred to as an eigensample). The module eigensample (ME) is a vector of length equal to the number of tumor markers, that captures the maximum amount of expression variation in a given module. It can also be interpreted as a weighted average of the expression values across the patients belonging to the module. The MEs of different modules can be correlated with each other to determine whether two highly correlated clusters should be merged. ME's with high correlation can be merged to reduce the patient network to a manageable number of modules using the mergeCloseModules function.

**Figure 2:**
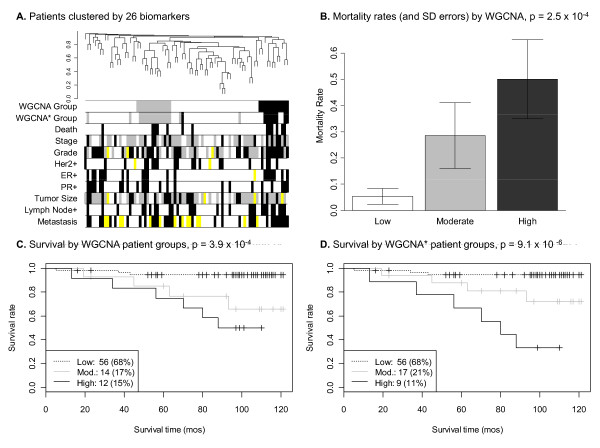
**Results of a WGCNA of 82 breast cancer patients and 26 markers**. **A**. Markers were clustered according to their expression levels across patient samples, so that each branch of the tree indicates a patient. The first row of white, grey and black colors below the tree indicates WGCNA patient groups that correspond to clusters of patients that have similar marker expression profiles. The second row consists of WGCNA* groups which is an approximation to WGCNA that relies on only three of the 26 markers. Subsequent rows consist of clinical variable data, where black matches with unfavorable prognostic factors, white is favorable, grey is intermediate, and yellow indicates missing data. Stage was coded as 1-3 with stage 1 colored white (there was one stage 4 patient that we re-coded as stage 3). Grade was coded as 1-3 with grade 1 colored white. Her2+, ER- and PR- were colored black. The presence of lymph node involvement (LNI) and metastasis were colored black. Tumor size was re-coded as quantiles, where tumors smaller than the 25^th ^percentile were colored white, tumors between the 25^th^-75^th ^percentiles were colored grey, and sizes greater than or equal to the 75^th ^percentile were colored black. **B-C**. WGCNA patient groups correspond to low, moderate (mod.) and high mortality. **D**. An approximation to the WGCNA groups "WGCNA*" that uses a subset of three markers (rather than the full marker set) is also highly related to patient survival.

Clinical variables can be visualized using color-bands underneath the dendrogram (cluster tree) to visually evaluate or refine merging parameters using plotDendroAndColors (see Figure 2A for an example). We optimized the module merging process to correspond with patient mortality. As a result, we identified three patient clusters with low, moderate and high mortality rates. Since our optimized module merging process may have overfit the data, we evaluated the prognostic accuracy of the resulting clusters in three independent data sets as described below.

#### 3. Evaluate the utility of WGCNA groups for survival prediction

To understand the clinical meaning of the three patient clusters (referred to as WGCNA groups) we studied the relationships between the groups and clinical variables. Conventional survival analysis methods such as Kaplan-Meier plots and log-rank tests were used to assess survival prediction, and we used a Kruskal-Wallis and Fisher's exact test to relate the WGCNA mortality groups to continuous and categorical clinical variables, respectively. We used a log rank test to confirm that the WGCNA patient groups were highly related to cancer survival, and then checked that these patient groups could not be exclusively defined by clinical variables (stage, grade, Her2+, ER+, PR+, tumor size, lymph node involvement and metastasis). We also evaluated survival prediction of the WGCNA groups while controlling for other predictive clinical variables in a multivariate Cox proportional-hazards model.

#### 4. Use classification trees to identify key markers for defining WGCNA groups

Depending on the number of markers analyzed, it may be practical to reduce the full marker set to a few key markers that would be more manageable in a diagnostic setting or validation analysis. After confirming that the WGCNA patient groups were predictive of survival in both univariate and multivariate analyses, we used recursive partitioning (or classification tree methodology) implemented in the rpart R function to identify a few markers that could approximate our WGCNA patient groups. The resulting approximate patient groups, or "WGCNA*" were related to clinical variables and evaluated in a multivariate Cox proportional-hazards model (as in step 3).

In summary, WGCNA and WGCNA* are both categorical grouping variables that attempt to classify low, moderate and high mortality risk groups according to their TMA marker expression data. The WGCNA variable uses data from the complete set of markers, and the WGCNA* variable approximates the WGCNA categories by identifying optimal thresholds for a small subset of these markers. Since a threshold can be defined in relation to its parent distribution, i.e. as a percentile, the WGCNA* classifier or "rule" can easily be evaluated in additional independent data sets.

### Application of WGCNA to a tumor expression array breast cancer data set

We applied the WGCNA methodology outlined above to a high-density TMA platform consisting of 26 putative tumor biomarkers measured on 82 breast cancer patients (Tables 1, 2). The patients were clustered by their expression profiles, which were transformed to adjacencies using the soft threshold (power) *β *= 10 and the signed network option for calculating adjacency in the WGCNA R package. The cutreeDynamic function was used (with options minClusterSize = 2 and deepSplit = 3) to generate 16 modules (not shown). Since we did not expect that these modules were robustly defined given the relatively small data set, we merged similar modules using the mergeCloseModules R function with the "cutHeight" parameter set to 0.15, which was optimal for obtaining fewer but more robustly defined modules that were significantly associated with survival (p-value = 3.9 × 10^-4^). Since the groups were defined with respect to the survival outcome, the p-value is overfit and should be interpreted as a descriptive (not inferential) measure. To arrive at an unbiased evaluation of the patient groups, we used independent gene expression data sets as described below. The WGCNA groups corresponded to mortality rates of 5.4%, 22%, and 50% (colored white, grey and black, respectively in Figure 2A, 2B, 2C).

**Table 1:** Summary statistics for trait data on 82 patients

Trait	Description
Tumor Size in cm	78 (5%)
Median (Range)	2.3 (0.2 - 9.0)
25th - 75th Quartile	1.5 - 3.0
Clinical Stage	82 (0%)
I	29 (35%)
II	36 (44%)
III-IV^a^	17 (21%)
Tumor Grade	79 (4%)
I	22 (27%)
II	23 (28%)
III	34 (41%)
Lymph Node+	29 (35%)
ER+	61 (74%)^b^
PR+	58 (71%)
HER-2/neu+	20 (24%)^c^
Metastasis+	32 (39%)^d^
# Deaths	13 (16%)
Time in months	82 (0%)
Median (Range)	97 (5, 121)
25th - 75th Quartile	70 - 110

^a^There was one stage IV patient.

^b^1, ^c^2 and ^d^14 missing observations.

Median and inter-quartile range (25^th ^- 75^th ^percentiles) are reported for skewed continuous variables. Categorical variables are reported as counts and percent total. The total number of observations and percentage of missing values are indicated adjacent to the variable name for continuous variables and categorical variables with more than two levels. Other missing values are indicated with footnotes.

**Table 2:** Summary statistics for TMA markers on 82 patients

TMA Marker	25th	Median	75th	# (% NA)
14-3-3 σ	5	33	63	79 (4%)
Annexin A1	0	0	6	82 (0%)
Beta-catenin	73	90	98	76 (7%)
BS106	0	3	30	79 (4%)
BU101	67	95	100	78 (5%)
MED28 expressed in the cytoplasm (cyt)	42	67	90	77 (6%)
MED28 expressed in the nucleus (nuc)	40	60	80	77 (6%)
Estrogen Receptor α (ER α)	2	18	50	78 (5%)
GATA3	27	79	100	82 (0%)
HER-2/neu	15	37	77	77 (6%)
Histone H3, acetylated on K4 (H3K4)	80	87	95	74 (10%)
Histone H3, acetylated on K18 (H3K18)	84	90	97	74 (10%)
Mammaglobin	17	57	83	80 (2%)
Na-K ATPase-α	33	58	80	75 (9%)
Na-K ATPase-β1	40	70	92	74 (10%)
p53	0	4	23	80 (2%)
Progesterone Receptor (PR)	0	15	57	78 (5%)
Prolactin inducible protein	0	0	1	79 (4%)
RIN-1	85	93	100	79 (4%)
Smad2	83	97	100	79 (4%)
Smad4 cyt	63	90	97	78 (5%)
Smad4 nuc	50	79	88	78 (5%)
TGF-β receptor II cyt	3	10	35	78 (5%)
TGF-β receptor II nuc	32	53	73	78 (5%)
S100A7	0	0	0	74 (10%)
Ying Yang 1 (YY1)	90	95	100	76 (7%)

Marker distributions are summarized by the median and inter-quartile range (25^th ^- 75^th ^percentiles). Number of observations and the percentage of missing values "% NA" are also provided.

**Figure 3:**
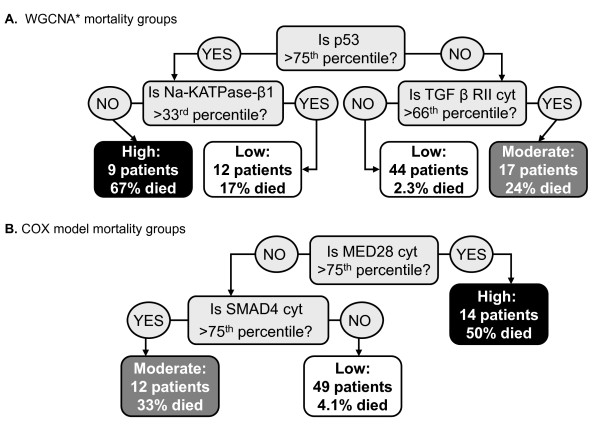
The WGCNA* and COX mortality group definitions. **A**. Classification trees were used to identify a subset of markers (3 out of 26 total) and their optimal thresholds for approximating the WGCNA groups. Nearly 88% (72 matches out of 82) of the mortality group assignments matched between WGCNA* and WGCNA. The markers and approximate thresholds included: p53 (dichotomized at the 75^th ^percentile), Na-KATPase-β1 (33^rd ^percentile) and TGF β receptor II (66^th ^percentile). High mortality was defined by high p53 and low Na-KATPase-β1. The group with a 17% mortality rate is called "low" because 10 of these 12 patients were assigned to the low mortality group by WGCNA. **B**. We also conducted a more traditional multimarker analysis by dichotomizing each of the 26 markers at an optimal threshold for survival prediction and then using a step-wise marker selection approach to achieve low, moderate and high mortality "COX" patient groups. This approach defined high mortality as high MED28, and moderate mortality as low MED28 and high Smad4. In both diagrams "cyt" indicates expression in the cytoplasm.

To test whether the median values of ordinal variables differed between WGCNA patient groups, we used the Kruskal Wallis test, which is a non-parametric multi-group comparison test. Boxplots were used to visualize the distribution for each group. Lymph node involvement, stage, metastasis and estrogen receptor positivity were significant at the 0.05 level, but none of these variables could completely define one or more of our patient groups (data not shown). Furthermore, an ordinal multivariate regression model predicting our patient groups from these four variables resulted in a McFadden pseudo R-square of only 0.068 (SPSS v16.0). After verifying that our WGCNA groups were distinct from our clinical variables, we evaluated its utility for survival prediction in the presence of other predictive variables. Variables that were significant at the 0.05 level in univariate Cox proportional-hazards models included lymph node involvement, stage, metastasis and Her2 positivity. A multivariate Cox proportional-hazards model that included these variables and our WGCNA variable found all predictors to be non-significant (p > 0.05) except the WGCNA mortality groups, where the high mortality group had a p-value of 0.037. These results suggest that the WGCNA mortality groups have distinct molecular characteristics that predict breast cancer survival independently of prognostic clinical variables. However, the WGCNA cluster variable was defined with respect to the 26 markers, which is cumbersome to validate. Therefore, we aimed to develop a simple classification rule (referred to as WGCNA*) which assigns each patient to its respective WGCNA cluster. Toward this end, we used classification trees implemented in the rpart R package, which automatically selects significant markers and corresponding thresholds. The classification tree led to a WGCNA* rule based on three markers p53, Na-KATPase-β1, and TGF β receptor II; with optimal thresholds corresponding to the 75^th^, 33^rd ^and 66^th ^percentiles, respectively (Figure 3A). Due to missing data in the p53, Na-KATPase-β1, and TGF β receptor II markers (2, 4 and 8 missing values, respectively), the WGCNA* rule initially resulted in only 66 patients with mortality group assignments. As a result, we confirmed that the missingness pattern of each marker was unrelated to survival and then replaced missing values by the median (rather than the average due to skewed distributions), so that all 82 patients were assigned to a WGCNA* mortality group. By construction, the WGCNA* mortality groups closely matched the original WGCNA groups, differing by only 10 patients (Table 3). As a result, the WGCNA* patient groups were highly predictive of patient survival (p-value = 9.1 × 10^-5^, Table 4), with mortality rates of 5.4%, 24%, and 67% (Figure 2D, 3A). The WGCNA* rule suggests that breast cancer patients with high p53 and low Na-KATPase-β1 have a high risk of death in comparison to other molecular profiles. Furthermore, patients with low p53 and high TGF β receptor II have a moderate mortality risk.

**Table 3:** Comparison of WGCNA* mortality group assignments to WGCNA and COX

WGCNA* Groups	WGCNA Groups			COX Groups		
	
	Low	Moderate	High	Low	Moderate	High
**Low**	51	1	4	40	7	3
**Moderate**	4	13	0	7	4	6
**High**	1	0	8	2	1	5

**Totals**	**N**	**Agree**	**Disagree**	**N**	**Agree**	**Disagree**
	
	82	72 (88%)	10 (12%)	75	49	26 (35%)

WGCNA* patient mortality group designations are compared with WGCNA and the COX definition, where diagonal elements indicate the number matches. Only 10 patients (12%) differed between WGCNA and WGCNA* in terms of their group designations, but 26 patients (out of 75, due to 7 unclassified patients by the COX definition) differed between WGCNA* and COX.

**Table 4:** Mortality comparison between WGCNA*, WGCNA and COX groups

Mortality Group	# Deaths/# Patients (% Mortality)		
	
	WGCNA	WGCNA*	COX
**Low**	3/56 (5.4%)	3/56 (5.4%)	2/49 (4.1%)
**Moderate**	4/14 (22%)	4/17 (24%)	4/12 (33%)
**High**	6/12 (50%)	6/9 (67%)	7/14 (50%)

**Log rank p**	3.9 × 10^-4^	9.1 × 10^-6^	1.6 × 10^-4^

The number of patients and their mortality rates were similar across WGCNA, WGCNA* and COX groups. The log rank test p-values were also similar, ranging from 3.9 × 10^-4 ^to 9.1 × 10^-6^.

**Figure 4:**
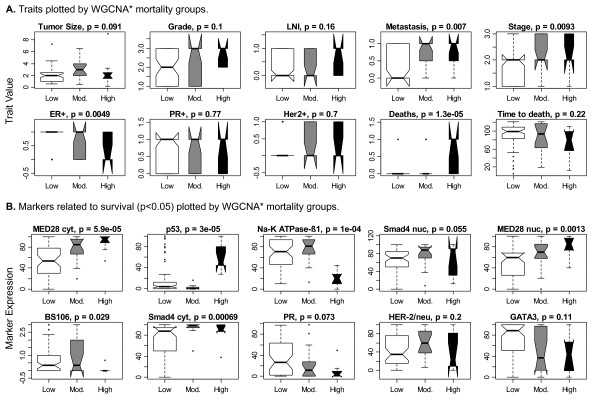
Variable and marker boxplots by WGCNA* mortality group. Kruskal-Wallis p-values are reported for the comparison of each variable and marker to the WGCNA* patient groups, where the WGCNA* patient groups are color coded to indicate low (white), moderate (grey) and high (black) mortality. **A**. Metastasis, stage, ER+ and death are significantly related to the WGCNA* groups (p < 0.05). **B**. The top 10 markers related to survival that achieved significance at p < 0.05 in a univariate Cox proportional-hazards model when dichotomized at an optimal cut-point. The boxplots indicate that no variable or marker by itself can define the WGCNA* groups. Abbreviations are as follows, "LNI" stands for Lymph Node Involvement, "cyt" indicates the TMA marker was expressed in the cytoplasm and "nuc" indicates nuclear expression.

To elucidate the clinical meaning of the WGCNA* groups, we related them to clinical variables. We found that the WGCNA* groups are significantly related to stage, metastasis and estrogen receptor positivity (p < 0.05, Figure 4A). A multivariate Cox proportional-hazards model that included the prognostic clinical variables (lymph node involvement, stage, metastasis and Her2+) had a slightly lower R^2 ^(0.306 versus 0.326) and hazard ratio (3.8 versus 5.9) for the high mortality group when WGCNA* was used as a predictor rather than the original WGCNA grouping variable based on all 26 markers (Table 5). However, these differences are negligible given that WGCNA* substantially reduced the number of markers needed for validation.

**Table 5:** Survival prediction of WGCNA, WGCNA* and COX groups in a multivariate Cox proportional-hazards (CPH) model

CPH Model Predictors	WGCNA		WGCNA*		COX	
	
	HR (CI)	p-value	HR (CI)	p-value	HR (CI)	p-value
Moderate Mortality	3.1 (0.5,19)	0.220	1.5 (0.3,7.3)	0.635	17.5 (1.7,178)	0.016
High Mortality	5.9 (1.1,31)	0.037	3.8 (0.8,18)	0.094	11.0 (1.2,102)	0.036
Lymph Node Involvement	0.9 (0.3,3.3)	0.900	1.1 (0.3,4.0)	0.879	1.2 (0.3,5.0)	0.767
Metastases	5.5 (0.4,72)	0.200	4.4 (0.3,58)	0.261	3.1 (0.2,59)	0.446
Stage	1.9 (0.5,7.7)	0.370	2.2 (0.5,8.9)	0.284	2.7 (0.4,17)	0.296
Her2+	2.9 (0.8,11)	0.120	3.0 (0.8,10)	0.091	1.7 (0.5,6.0)	0.433

**# Observations**	66		66		60	
**Model R^2 ^(p-value)**	0.326 (1.2 × 10^-4^)		0.306 (1.1 × 10^-4^)		0.386 (4.7 × 10^-5^)	

Hazard ratios (HR) and their 95% confidence intervals (CI) are reported for each model along with coefficient p-values. WGCNA and COX patient mortality groups predicted survival at p < 0.05, and the overall model R^2 ^and p-values were similar across all three models. The COX model achieved the strongest hazards ratios for the moderate and high mortality groups. Her2+ was the strongest variable predictor but did not achieve significance at the 0.05 level. Variables were selected for multivariate analysis if they were significantly related to survival in a univariate CPH model (p < 0.05).

We verified that no single marker could define the WGCNA groups (Figure 4B). Cox proportional-hazards models that included WGCNA* and each marker individually (dichotomized at its optimal survival prediction threshold) found WGCNA* to be the top predictor in all cases (data not shown).

### Comparison of WGCNA* patient groups with a conventional step-wise analysis

While WGCNA defines patient mortality groups that predict survival independently of other clinical variables, it is interesting to know how this approach compares with a more conventional stepwise variable selection approach [37-39]. We analyzed the same TMA data by first dichotomizing each marker at an optimal threshold for survival prediction. Ten markers could be dichotomized at a level that achieved a minimum of five patients per group and a univariate Cox proportional-hazards model p < 0.05. We included these 10 markers in a multivariate Cox proportional-hazards model and removed the least significant predictor in a step-wise manner, until all remaining variables achieved significance at the 0.05 level. Four markers: MED28 expressed in the cytoplasm, p53, Smad4 expressed in the cytoplasm and Her2 were retained. The resulting model had an R^2 ^of 0.321. We then used classification trees (rpart with complexity parameter 0.1) to identify a subset of markers and their thresholds for defining low, moderate and high mortality patient groups. This was achieved with two markers MED28 and Smad4, dichotomized at their 75^th ^percentiles, where high MED28 resulted in high mortality and low MED28 in conjunction with high Smad4 resulted in moderate mortality (Figure 3B). The resulting "COX" patient groups were significantly related to survival (p = 1.6 × 10^-4^) and had mortality rates of 4.1%, 33% and 50%. While the COX variable had similar mortality rates to WGCNA and WGCNA*, there was a substantial 35% difference in the assignment of patients to mortality groups (Table 3, Additional File 1). A multivariate Cox proportional-hazards model that included the COX variable and the prognostic clinical variables, found the COX variable to be the best predictor with p-values of 0.016 and 0.036 for the moderate and high mortality groups, respectively (Table 5). Finally, to directly compare the WGCNA* and COX variables, we included both in a Cox proportional-hazards model with and without the prognostic clinical variables. The WGCNA* high mortality group was the most significant predictor (p = 0.017) in the absence of clinical variable data, but when prognostic clinical variables were included, the COX moderate and high mortality groups were the only significant predictors in the model.

In summary, the WGCNA* and COX mortality groups are distinct from each other and are both important predictors of breast cancer survival in our TMA data. While COX outperforms WGCNA* in the presence of prognostic clinical variables, it was also created by optimizing the significance of its underlying markers in a Cox proportional-hazards model. Thus, the COX variable's superior performance could possibly be explained by over-fitting in our TMA data set and may not validate in other data sets. To test this, we attempted to validate the WGCNA* and COX mortality group rules in independent gene expression data sets.

### Validation analysis of WGCNA* and COX groups in gene expression data sets

We applied the WGCNA* and COX rules to three independent Affymetrix HG-U133A data sets (GSE3494, GSE1456, GSE2990) [24-26]. After data cleaning (as described in the Methods section) the data sets consisted of 207, 146 and 173 breast cancer patients for the Miller 2005, Pawitan 2005 and Sotiriou 2006 data sets, respectively (Additional File 2A). The data sets had mortality rates ranging from 17%-27%, with the Pawitan 2005 and Sotiriou 2006 data sets being most similar to the 16% rate in our TMA data. Other than survival, grade was the only clinical variable common to all three data sets, and it appeared fairly consistent across the gene expression and TMA data sets. The probe set distributions were also consistent, where the interquartile ranges overlapped for all Miller 2005 and Pawitan 2005 probe sets; which overlapped with Sotiriou 2006 in most cases (Additional File 2B). There were no anomalies to disqualify a data set or probe set for validation analysis.

**Figure 5:**
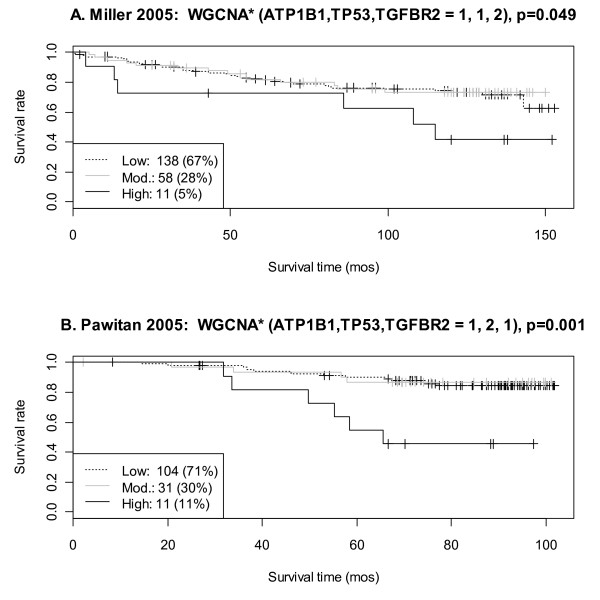
Validation of the WGCNA* high mortality group in two independent gene expression data sets (A-B). **A**. Results for the Miller 2005 data set are shown for the following probe sets ATP1B1: 201242_s_at, TP53: 201746_at, and TGFBR2: 208944_at. The Pawitan 2005 data set validated for all probe set combinations, but results for ATP1B1: 201242_s_at, TP53: 211300_s_at, and TGFBR2: 207334_s_at are shown in **B**. Data set information can be found in Additional File 2, and additional validation results can be found in Table 6.

On the Affymetrix HG-U133A array, the protein Na-KATPase-β1 was represented by gene ATP1B1 which had two probe sets 201242_s_at and 201243_s_at. Similarly, p53 corresponded to the TP53 gene (201746_at and 211300_s_at); and TGF β receptor II corresponded to TGFBR2 (207334_s_at and 208944_at). Thus there were eight versions of the WGCNA* rule corresponding to each probe set combination (2 × 2 × 2). Similarly for the COX rule, the proteins MED28 cyt and Smad4 cyt were represented by genes MED28 (214831_at, 218438_s_at) and SMAD4 (202526_at, 202527_s_at) resulting in four versions of the COX rule per validation data set. The strongest WGCNA* validation was achieved in the Pawitan 2005 data set, where all eight probe set combinations validated for the high mortality group (p < 0.1, Table 6, Figure 5). In the Miller 2005 data set, two probe set combinations validated. No probe sets combinations validated in the Sotiriou 2006 data set. Applying the same validation criteria (p < 0.1), the COX rule did not validate in any data set for any probe set combination (data not shown). In summary, the WGCNA* high mortality group validated in two of three Affymetrix breast cancer data sets, suggesting that patients with high TP53 and low ATP1B1 may have a worse prognosis than patients with other profiles. Since high p53/TP53 is a well known indicator of poor prognosis, the following section verifies that the combination of Na-KATPase-β1/ATP1B1 and p53/TP53 in both the protein and gene expression data sets is a stronger survival predictor than p53/TP53 alone.

**Table 6:** Validation results for WGCNA* mortality groups in three Affymetrix data sets

#	Data Set	Marker Probeset			Hazard Ratio		P-values			Validates at 0.1
			
		ATP1B1	TP53	TGFBR2	**Mod**.	High	**Mod**.	High	Model	
1	Miller 2005	1	1	1	0.88	2.35	0.693	0.053	0.173	yes
**2**	**Miller 2005**	**1**	**1**	**2**	**0.94**	**2.39**	**0.853**	**0.049**	**0.184**	**yes**
3	Miller 2005	1	2	1	1.10	0.93	0.777	0.884	0.944	no
4	Miller 2005	1	2	2	1.17	0.94	0.611	0.914	0.865	no
5	Miller 2005	2	1	1	0.82	1.18	0.538	0.750	0.754	no
6	Miller 2005	2	1	2	0.88	1.20	0.676	0.727	0.840	no
7	Miller 2005	2	2	1	1.05	0.32	0.873	0.258	0.371	no
8	Miller 2005	2	2	2	1.12	0.32	0.709	0.265	0.351	no
9	Pawitan 2005	1	1	1	0.64	3.17	0.429	0.024	0.062	yes
10	Pawitan 2005	1	1	2	0.41	2.91	0.158	0.036	0.026	yes
**11**	**Pawitan 2005**	**1**	**2**	**1**	**0.93**	**4.89**	**0.894**	**0.001**	**0.014**	**yes**
12	Pawitan 2005	1	2	2	0.41	4.05	0.156	0.004	0.004	yes
13	Pawitan 2005	2	1	1	0.64	3.22	0.430	0.023	0.060	yes
14	Pawitan 2005	2	1	2	0.41	2.95	0.159	0.034	0.025	yes
15	Pawitan 2005	2	2	1	0.92	4.37	0.880	0.002	0.022	yes
16	Pawitan 2005	2	2	2	0.41	3.62	0.152	0.008	0.007	yes

The Pawitan 2005 data set validated at the 0.1 level for all eight probe set combinations for the high mortality group. The Miller 2005 data set validated for two probe set combinations. The WGCNA* moderate mortality group did not validate. None of the probe set combinations validated for the Sotiriou 2006 data set (not shown). The rows highlighted bold indicate probe set combinations with Kaplan-Meier plots in Figure 5. Probe set abbreviations are as follows ATP1B1: 1 = 201242_s_at, 2 = 201243_s_at; TP53: 1 = 201746_at, 2 = 211300_s_at; TGFBR2: 1 = 207334_s_at, 2 = 208944_at.

### A comparison of p53 and the WGCNA* high mortality group

Since the WGCNA* high mortality group was defined by low Na-KATPase-β1 and high p53, we checked whether p53 alone would be a sufficient or possibly superior survival predictor. In our TMA data, the optimal dichotomized threshold for p53 was the 75^th ^percentile. In a Cox proportional-hazards model that included both the WGCNA* high mortality group (coded as high versus moderate and low combined) and the dichotomized p53 marker, the hazards ratio for the WGCNA* high mortality group was more than two-fold higher at 4.5 (p = 0.07) versus a hazards ratio of 2.1 (p = 0.38) for the dichotomized p53 marker. In the gene expression data, the continuous form of the TP53 variable was not significant while the WGCNA* high mortality group maintained significance at the 0.05 level for all 8 of the Pawitan 2005 models. The dichotomized TP53 marker did achieve significance at the 0.05 level in two of the Miller 2005 models, but high TP53 indicated a protective effect, which is inconsistent with current (protein-level) findings (HR = 0.35 and p = 0.04 for both models). In summary, low Na-KATPase-β1 (< 33^rd ^percentile) in combination with high p53 (>75^th ^percentile) is a stronger predictor of mortality than p53 alone in both our TMA data and the Pawitan 2005 gene expression data set.

### Analysis of the WGCNA* high mortality group in the Pawitan 2005 data set

Since the WGCNA* high mortality group consistently validated in the Pawitan 2005 data set, we explored the relationship between this group and the available Pawitan 2005 variables: subtype (Basal, ERBB2, luminal A, luminal B and normal like) and grade (I-III). In this data set, the high mortality group consisted of 11 patients, 7 of which were luminal B, one luminal A, one basal, and two were missing subtypes. Thus, subtype was significantly related to WGCNA* high mortality (Fisher's exact test p = 1.5 × 10^-4^). Similarly, seven of the high mortality group patients were grade 3, two were grade 2, and two were grade 1, although this relationship did not achieve significance (Fisher's exact test p = 0.194). In a multivariate Cox proportional-hazards model with subtype coded as luminal B versus other types and grade coded as an ordinal variable, the WGCNA* high mortality group was the strongest predictor with a hazards ratio of 4.22 (95% CI: 1.3, 14.1, p = 0.019). See Additional File 3 for characteristics of the WGCNA* mortality groups.

## Discussion

TMA data are typically used to test hypotheses regarding the prognostic value of tumor markers. Here we use breast cancer TMA data to demonstrate that TMA data are also valuable for tumor class discovery. We show that using weighted correlation networks analysis for clustering patients across 26 TMA markers identifies patient groups that have distinct molecular profiles associated with mortality. The WGCNA mortality groups were significantly associated with survival even after controlling for stage, metastasis, lymph node involvement and Her2 positivity in a multivariate Cox regression model. Since measuring 26 tumor markers may be impractical, we used a classification tree predictor to find a close approximation (referred to as "WGCNA*") of the WGCNA mortality groups. The resulting classification rule "WGCNA*" relied on only three TMA markers: p53, Na-KATPase-β1, and TGF β receptor II. The WGCNA* mortality groups with low (5.4%), moderate (24%) and high (67%) mortality rates differed by 35% from a mortality classification developed by a more traditional step-wise Cox regression approach. The WGCNA* mortality classification validated in two out of three independent Affymetrix gene expression data sets, while the traditional Cox regression classification did not validate.

Our three markers are not included in major commercial gene expression marker sets that predict breast tumor recurrence such as MammaPrint (70 markers) or Oncotype DX (16 markers) [40,41], although TGF β receptor II (TGFBR2) was among the initial set of 250 candidate genes considered by Oncotype DX. To our knowledge there are no additional reports of these three markers in combination (or pair-wise combinations) in the breast cancer literature. Turning to single marker studies, our major low mortality group defined by p53 ≤ 75^th ^percentile and TGF β receptor II ≤ 66^th ^percentile is consistent with literature results for p53, as increased p53 expression has been implicated in poor breast cancer prognosis [42], but potentially inconsistent with results for TGF β receptor II since loss of TGF β receptor II function has been implicated in breast cancer metastasis [43]. There is limited breast cancer literature on Na-KATPase-β1, but elevated levels were found in African American breast cancer cells in comparison to Caucasian, where the former cancer is typically more aggressive [44]. If one interprets this to mean that elevated Na-KATPase-β1 expression is associated with poorer prognosis, it would be inconsistent with our finding that the high mortality group has low Na-KATPase-β1 (p53 > 75^th ^percentile and Na-KATPase-β1 ≤ 33^rd ^percentile). While the limited congruence between our multimarker mortality rule and the single marker studies may be partly explained by differences in patients, outcome variables, and high/low expression definitions; it is also likely due to the additional information gained by analyzing marker combinations. Thus, a validation of our results would best be achieved in an analogous multimarker setting.

Our study has several strengths and limitations. Here we have shown that correlation network methodology is useful for defining patient groups based on multiple tumor markers. The methods described here handle 10's to 10000's of tumor markers, and should be useful for other multimarker TMA studies. Furthermore, we have identified three tumor markers, p53, Na-KATPase-β1, and TGF β receptor II that predict breast cancer survival in our TMA data set and in two independent gene expression data sets. However, we acknowledge the following limitations. First, our three marker mortality rule was developed on only one TMA data set, and it should be validated in other TMA data sets. Second, our analysis was restricted to 26 available prognostic markers. These markers neither represented a comprehensive set of tumor markers, nor were randomly selected from a comprehensive tumor marker set. Rather, they had been acquired for use in other oncology studies. As more markers become available, the mortality group definition could improve and the WGCNA* definition may change. However, it would be easy to incorporate additional data as WGCNA can handle large data sets with thousands of markers and/or samples. Finally, the moderate WGCNA* mortality group did not validate in the gene expression data. While this could be due to RNA and protein expression level differences, additional data is needed to support TGF β receptor II as a prognostic marker.

## Conclusions

Weighted correlation network analysis identifies patient mortality groups that cannot be defined by a single marker or clinical variable and are highly related to breast cancer survival. The p53, Na-KATPase-β1, and TGF β receptor II markers may be useful in a clinical setting for predicting breast cancer survival.

## Abbreviations

TMA: tissue microarray; ER: estrogen receptor; PR: progesterone receptor; LVI: lymphovascular invasion; HR: hazard ratio; CI: confidence interval; WGCNA: weighted gene correlation network analysis.

## Competing interests

TThe authors declare that they have no competing interests.

## Authors' contributions

APP and SH wrote the manuscript. APP conducted the analysis in collaboration with NKY, SH developed the WGCNA methodology and supervised the analysis. LB processed the microarray data sets. VM, MA, EM and AR collected the TMA data. LG and DC provided biological direction. All authors read and approved the final manuscript.

## Pre-publication history

The pre-publication history for this paper can be accessed here:

http://www.biomedcentral.com/1471-2407/11/230/prepub

## Supplementary Material

Additional File 1 Patients clustered by 26 biomarkers and colored by WGCNA, WGCNA* and COX groups. The WGCNA and WGCNA* groups are similar in terms of their assignments of patients to low (white), moderate (grey) and high mortality (black) groups. In comparison, the COX groups defined by a more traditional approach (step-wise cox model selection) were quite different. Yellow indicates missing values. Click here for file

Additional File 2 **Summary statistics for traits and markers from three gene expression data sets. A**. The Pawitan 2005 and Sotiriou 2006 data sets were most similar to our TMA data in terms of the percent mortality and survival times, which was 16% and 8 years in our TMA data and 17-22% and 7 years in the Pawitan 2005 and Sotiriou 2006 data sets. The Miller 2005 data set had a longer follow-up time which may explain its higher mortality rate (27%). Estrogen receptor positivity was similar across studies (74%-87%) while progesterone receptor positivity differed by 37% between the Miller 2005 data set and our TMA data. **B**. Marker expression data for HG-U133A probe sets that best matched our TMA marker data, where each of our TMA markers were represented by two probe set IDs. Medians are plotted with interquartile range (IQR) error bars. Distributions were similar (IQR's overlapped) for at least one of the two probe sets for each marker. Click here for file

Additional File 3 Summary statistics for variables by WGCNA* mortality group. Median and inter-quartile range (25^th ^- 75^th ^percentiles) are reported for skewed continuous variables. Categorical variables are reported as counts and percent total. The total number of observations and percentage of missing variable data are indicated adjacent to the variable name for continuous variables and categorical variables with more than two levels. Other missing variable data are indicated with footnotes. Click here for file
