# Supplementary material for: Protein expression based multimarker analysis of breast cancer samples
Source: BMC Cancer. 2011 Jun 8;11:230. doi: 10.1186/1471-2407-11-230 (PMC3142534; doi:10.1186/1471-2407-11-230)

Additional File 1. Patients clustered by 26 biomarkers and colored by WGCNA, WGCNA* and COX groups. The WGCNA and WGCNA* groups are similar in terms of their assignments of patients to low (white), moderate (grey) and high mortality (black) groups. In comparison, the COX groups defined by a more traditional approach (step-wise cox model selection) were quite different. Yellow indicates missing values.

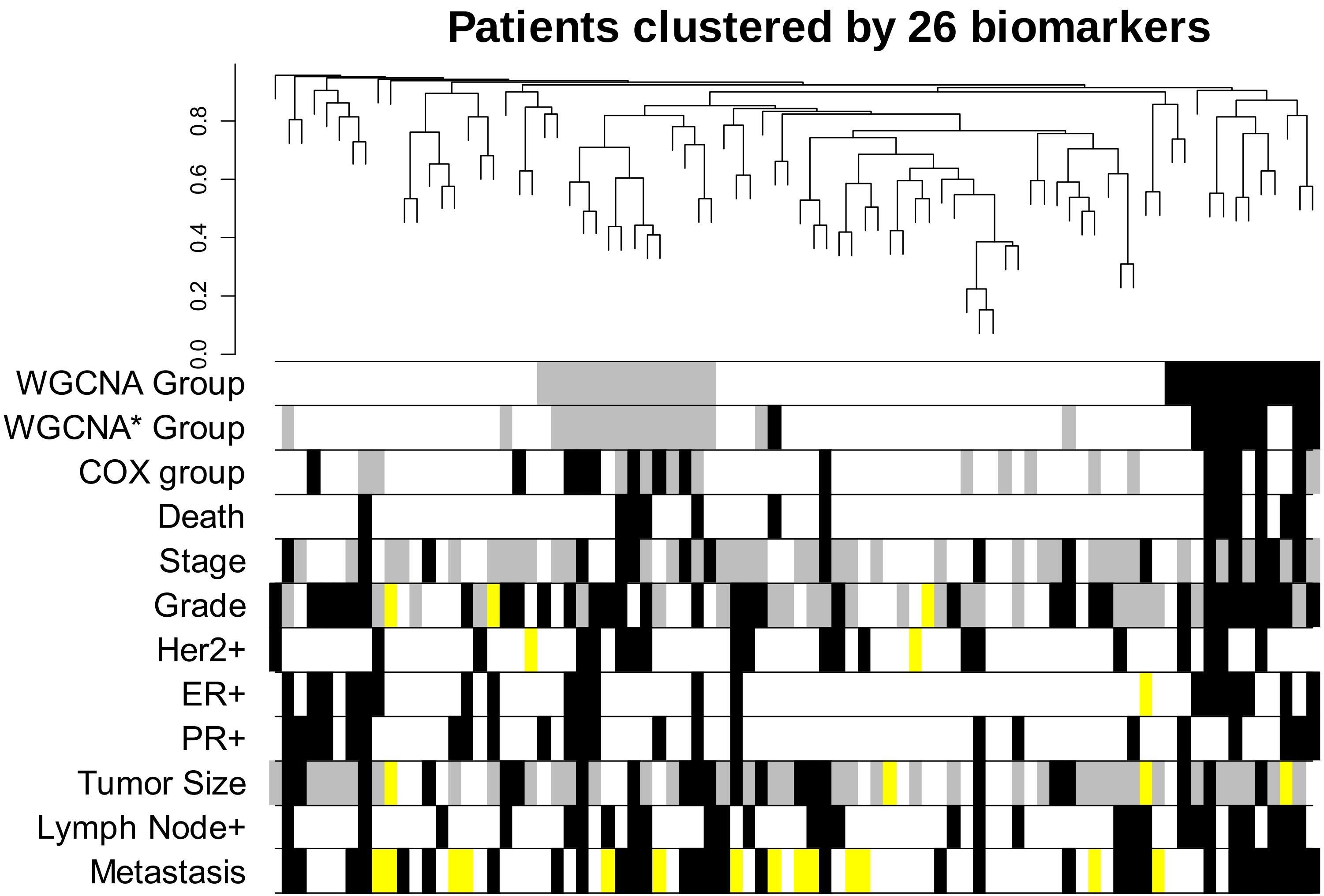

Supplement: Additional File 1 — Patients clustered by 26 biomarkers and colored by WGCNA, WGCNA* and COX groups. The WGCNA and WGCNA* groups are similar in terms of their assignments of patients to low (white), moderate (grey) and high mortality (black) groups. In comparison, the COX groups defined by a more traditional approach (step-wise cox model selection) were quite different. Yellow indicates missing values. [file 1471-2407-11-230-S1.PDF]
