# Supplementary material for: Protein expression based multimarker analysis of breast cancer samples
Source: BMC Cancer. 2011 Jun 8;11:230. doi: 10.1186/1471-2407-11-230 (PMC3142534; doi:10.1186/1471-2407-11-230)

Additional File 2. Summary statistics for traits and markers from three gene expression data sets. A. The Pawitan 2005 and Sotiriou 2006 data sets were most similar to our TMA data in terms of the percent mortality and survival times, which was 16% and 8 years in our TMA data and 17-22% and 7 years in the Pawitan 2005 and Sotiriou 2006 data sets. The Miller 2005 data set had a longer follow-up time which may explain its higher mortality rate (27%). Estrogen receptor positivity was similar across studies (74%-87%) while progesterone receptor positivity differed by 37% between the Miller 2005 data set and our TMA data. **B.** Marker expression data for HG-U133A probe sets that best matched our TMA marker data, where each of our TMA markers were represented by two probe set IDs. Medians are plotted with interquartile range (IQR) error bars. Distributions were similar (IQR's overlapped) for at least one of the two probe sets for each marker.

A. Trait Data	Gene Expression Study		
	Miller 2005	Pawitan 2005	Sotiriou 2006
GEO ID	GSE3494	GSE1456	GSE2990
Total # Obs	207	146	173
% Mortality	27% (55)	17% (25)	22% (38)
Survival Time	120 (53,130)	85 (69, 94)	85 (41,119)
Grade	205	135	156
I	25% (52)	20% (27)	38% (59)
II	52% (106)	41% (55)	30% (47)
III	23% (47)	39% (53)	32% (50)
ER+	87% (177)[a]	-	80% (136)[b]
PR+	34% (68)	-	-
Age	65 (52,73)	-	57 (46, 64)
Tumor Size	2 (1.5, 2.6)	-	2 (1.4, 2.5)
Lymph Node+	34% (68)[c]	-	15% (26)[a]
Subtype	-	127	-
Basal	-	12% (15)	-
ERBB2	-	12% (15)	-
Luminal A	-	30% (38)	-
Luminal B	-	17% (22)	-
Normal Like	-	29% (37)	-

[a]3, [b]4 and [c]9 missing values.

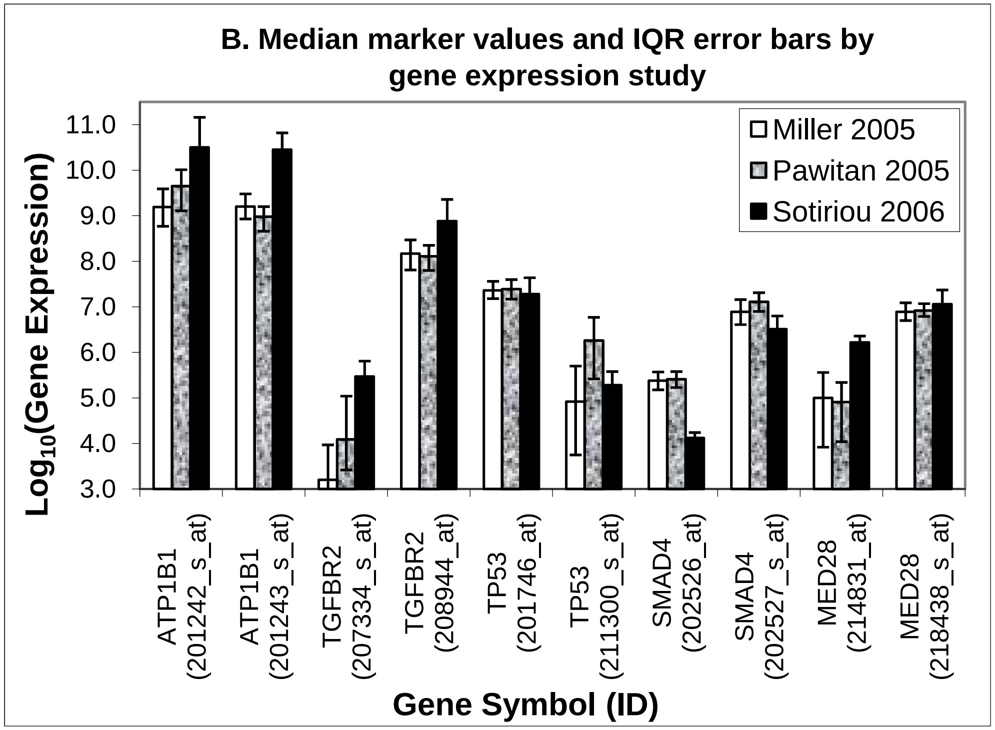

Supplement: Additional File 2 — Summary statistics for traits and markers from three gene expression data sets. A. The Pawitan 2005 and Sotiriou 2006 data sets were most similar to our TMA data in terms of the percent mortality and survival times, which was 16% and 8 years in our TMA data and 17-22% and 7 years in the Pawitan 2005 and Sotiriou 2006 data sets. The Miller 2005 data set had a longer follow-up time which may explain its higher mortality rate (27%). Estrogen receptor positivity was similar across studies (74%-87%) while progesterone receptor positivity differed by 37% between the Miller 2005 data set and our TMA data. B. Marker expression data for HG-U133A probe sets that best matched our TMA marker data, where each of our TMA markers were represented by two probe set IDs. Medians are plotted with interquartile range (IQR) error bars. Distributions were similar (IQR's overlapped) for at least one of the two probe sets for each marker. [file 1471-2407-11-230-S2.PDF]
