# Supplementary material for: Protein expression based multimarker analysis of breast cancer samples
Source: BMC Cancer. 2011 Jun 8;11:230. doi: 10.1186/1471-2407-11-230 (PMC3142534; doi:10.1186/1471-2407-11-230)
Supplement: Additional File 3 — Summary statistics for variables by WGCNA* mortality group. Median and inter-quartile range (25th - 75th percentiles) are reported for skewed continuous variables. Categorical variables are reported as counts and percent total. The total number of observations and percentage of missing variable data are indicated adjacent to the variable name for continuous variables and categorical variables with more than two levels. Other missing variable data are indicated with footnotes. [file 1471-2407-11-230-S3.PDF]

Additional File 3. Summary statistics for variables by WGCNA* mortality group. Median and inter-quartile range (25th - 75th percentiles) are reported for skewed continuous variables. Categorical variables are reported as counts and percent total. The total number of observations and percentage of missing variable data are indicated adjacent to the variable name for continuous variables and categorical variables with more than two levels. Other missing variable data are indicated with footnotes.

**Trait**	**Low (N=56)**	**Moderate (N=17)**	**High (N=9)**
Tumor Size in cm	52 (7%)	17 (0%)	9 (0%)
Median (Range)	2.0 (0.6 – 7.3)	3.0 (0.5 – 6.5)	2 (0.2 – 9.0)
25th – 75th Quartile	1 – 2.5	2.0 – 4.0	1.5 – 2.5
Clinical Stage	56 (0%)	17 (0%)	9 (0%)
I	24 (43%)	3 (18%)	2 (22%)
II	26 (46%)	7 (41%)	3 (33%)
III-IV	6 (11%)	7 (41%)	4 (44%)
Tumor Grade	53 (5%)	17 (0%)	9 (0%)
I	17 (30%)	5 (29%)	0
II	17 (30%)	3 (18%)	3 (33%)
III	19 (34%)	9 (53%)	6 (67%)
Lymph Node+	16 (29%)	8 (47%)	5 (56%)
ER+	46 (84%)[a]	12 (71%)	3 (33%)
PR+	41 (73%)	11 (65%)	6 (67%)
HER-2/neu+	12 (22%)[b]	5 (29%)	3 (33%)
Metastasis+	15 (33%)[c]	11 (73%)b	6 (75%)[a]
# Deaths	3 (5%)	4 (24%)	6 (67%)
Time in months	56 (0%)	17 (0%)	9 (0%)
Median (Range)	98.5 (5 – 121)	93 (19 – 121)	80 (13 – 110)
25th – 75th Quartile	85 – 108.5	63 – 116	56 – 99

[a]1, [b]2 and [c]11 missing observations.
